# Comprehensive analysis of cancer hallmarks in cutaneous melanoma and identification of a novel unfolded protein response as a prognostic signature

**DOI:** 10.18632/aging.103974

**Published:** 2020-10-26

**Authors:** Qi Wan, Lin Jin, Zhichong Wang

**Affiliations:** 1 State Key Laboratory of Ophthalmology, Zhongshan Ophthalmic Center, Sun Yat-Sen University, Guangzhou 510064, China

**Keywords:** UPR-related genes, cancer hallmarks, prognostic biomarker, melanoma

## Abstract

Molecular pathways regulating the initiation and development of melanoma are potential therapeutic targets for this aggressive skin cancer. Therefore, transcriptome profiles of cutaneous melanoma were obtained from a public database and used to systematically evaluate cancer hallmark pathways enriched in melanoma. Finally, the unfolded protein response pathway was screened out, and the unfolded protein response-related genes were used to develop a robust biomarker that can predict the prognosis of melanoma, especially for younger, metastatic and high Clark level patients. This biomarker was further validated in two other independent datasets. In addition, melanoma patients were divided into high- and low-risk subgroups by applying a risk score system. The high-risk group exhibited higher immune infiltration and higher expression of N6-methyladenosine RNA methylation regulators, and had significantly shorter survival times than the low-risk subgroup. Gene Set Enrichment Analysis revealed that, among the enriched genes, gene sets involved in immune response and the extracellular matrix receptor interaction were significantly activated in the high-risk group. Our findings thus provide a new clinical application for prognostic prediction as well as potential targets for treatment of melanoma.

## INTRODUCTION

Melanoma is a severely life-threatening type of skin cancer with high malignant metastasis [1]. About 75% of deaths in skin cancer are caused by melanoma which had become one of the most difficult human cancers to cure. Once it has spread metastasized, physical therapy is difficult to work and the 5 years survival rate will drop to 20% from 99% [2–5]. Thus, it is urgently required to explore the new prognostic pathways and signatures of melanoma to improve prognosis and guide more effective treatment.

Melanoma originates from the malignant transformation of melanocytes. The melanocytes will produce large number of melanin when stimulated by environmental factors, such as ultraviolet rays. Afterwards, benign melanocytes assemble clusters or format nevi because of uneven distribution of melanin. Although most of the changes are benign, combined the influence of environmental and genetic risk factors, this transformation will lead to cutaneous melanoma at some degree [6]. The exact steps that lead to initiation of melanoma still remain undefined. For example, the research for genetic evolution of melanoma found that only a third of melanomas appears to be associated with a pre-existing nevi [7, 8]. Whether there exist different biological signaling regulations between melanoma and nevi is not clear. However, gene mutation result in genetic diversity and susceptibility to DNA damage stimulated by ultraviolet light were the obviously definitive reasons [9]. Genomic technologies analysis has proved several genetic mutations and correlated pathways are closely associated with melanoma initiation and progression [10, 11]. For instance, mutations of BRAF and NRAS target the mitogen-activated protein kinase (MAPK) pathway which is disorderly regulated in almost all melanomas [12, 13]. MAPK pathway mainly associated with cell proliferation, but various other downstream regulations will cause tumor metastasis and cellular metabolic disorders [14]. Moreover, the mutation of CDKN2A leads HDM2 or MDM2 to inactivate p53 pathway which cause p53 loss and increase the survival of tumor cells [15–17]. Thanks to these basic researches, the emergence and approval of inhibitors like BRAF, RAS and MEK bring a promising treatment for melanoma patients [18, 19]. Therefore, it’s not surprising that gene biomarkers and molecular pathways regulating melanoma can provide us with more attractive therapeutic targets for this aggressive cancer.

Although there have been increasing studies exploring the melanoma by using different individual risk gene and pathway, a comprehensive analysis with an overall landscape of cancer hallmark pathways and related genes is still lacking [20–22]. Fortunately, the availability of public, large-scale datasets like the cancer genome atlas (TCGA) and Gene Expression Omnibus (GEO) databases which afforded numerous transcriptome profiles to investigate potential cancer hallmark pathways enriched in melanoma and the novel correlated gene features that can predict the clinical outcomes of patients.

Therefore, in this research, we first extracted 50 cancer hallmark pathways from the molecular signature database (MSigDB) and systematically evaluated the differential activities of pathways in multiple datasets. Next, we used univariate and multivariate cox regression method to identify several prognostic hallmark pathways as well as unfolded protein response related features associated with the clinical characteristics. We found that the expression of unfolded protein response feature plays critical roles in the prognostic process of melanoma and could be an independence potential biomarker.

## RESULTS

### The landscape of cancer hallmark pathways in melanoma

**Figure 1:**
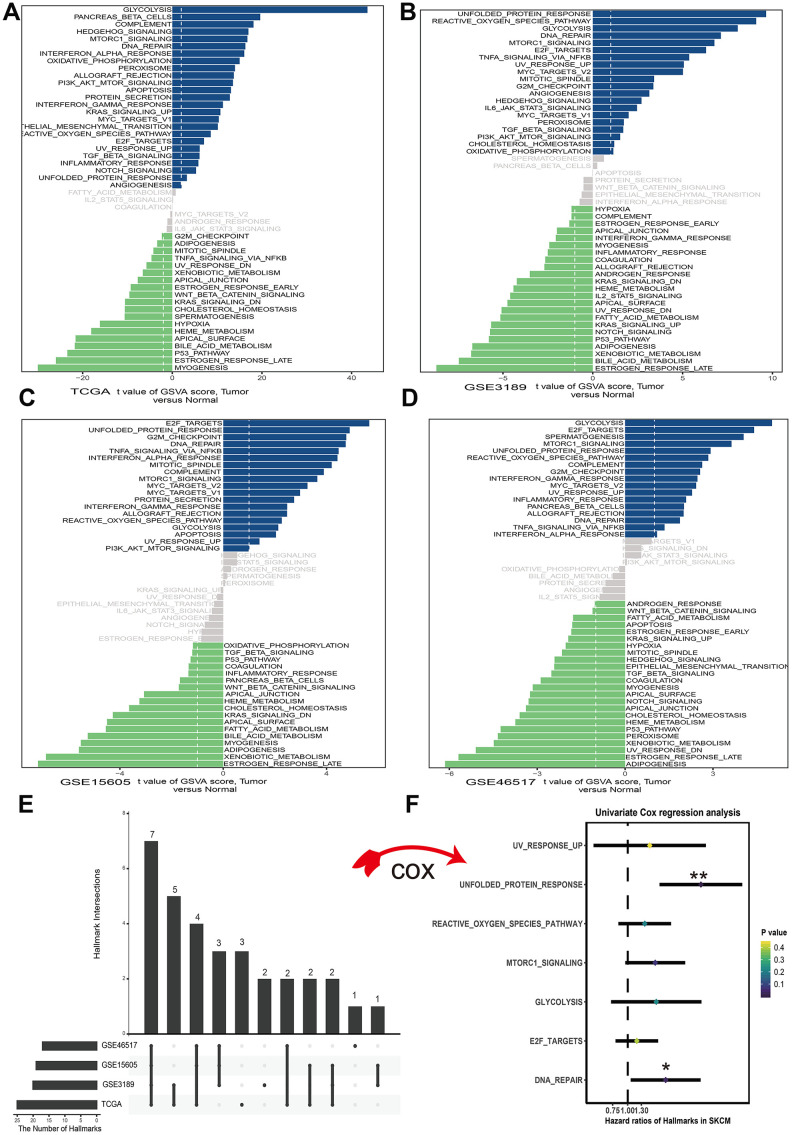
**Differences in cancer hallmark pathway activities between melanoma and normal sample scored by GSVA method.** (**A**) Cancer hallmark pathways in TCGA dataset. (**B**) Cancer hallmark pathways in GSE3189 dataset. (**C**) Cancer hallmark pathways in GSE15605 dataset. (**D**) Cancer hallmark pathways in GSE46516 dataset. The blue bars stand for the up-regulated pathways and the green bars mean down-regulated pathways. The x-axis is the t value of GSVA score. (**E**) Upset plot of different cancer hallmarks in multiple datasets. The dark bar on the left of drawing represents the amount of each dataset. The dark dots in the matrix at right of drawing represent the intersections of cancer hallmarks. (**F**) Forest plots of 7 cancer hallmarks, among which only unfolded protein response and DNA repair significantly correlated with OS (Overall survival) in TCGA dataset. *p<0.05; **p<0.01.

To assess the cancer hallmark pathways enriched in tumor group, we performed gene set variation analysis in four datasets which including GSE3189, GSE15605, GSE46517 and TCGA. According to the cutoff criteria, 25 hallmark pathways differentially activated in TCGA dataset (Figure 1A), 21 hallmark pathways significantly increased in GSE3189 (Figure 1B), 19 hallmark pathways actively expressed in GSE15605 (Figure 1C) and 17 hallmark pathways increasingly expressed in GSE46517 (Figure 1D). The Upset plot showed that there were 7 cancer hallmark pathways commonly enriched in tumor type among four melanoma datasets (Figure 1E). These pathways contained UV response up, unfolded protein response, reactive oxygen species pathway, mTORC1 signal, glycolysis, E2F targets and DNA repair among which only unfolded protein response (p<0.01) and DNA repair (p<0.05) significantly correlated with OS in TCGA dataset (Figure 1F). Finally, unfolded protein response regarded as the most significant pathway in melanoma and selected for further research.

### Differentially expressed UPRRGs

**Figure 2:**
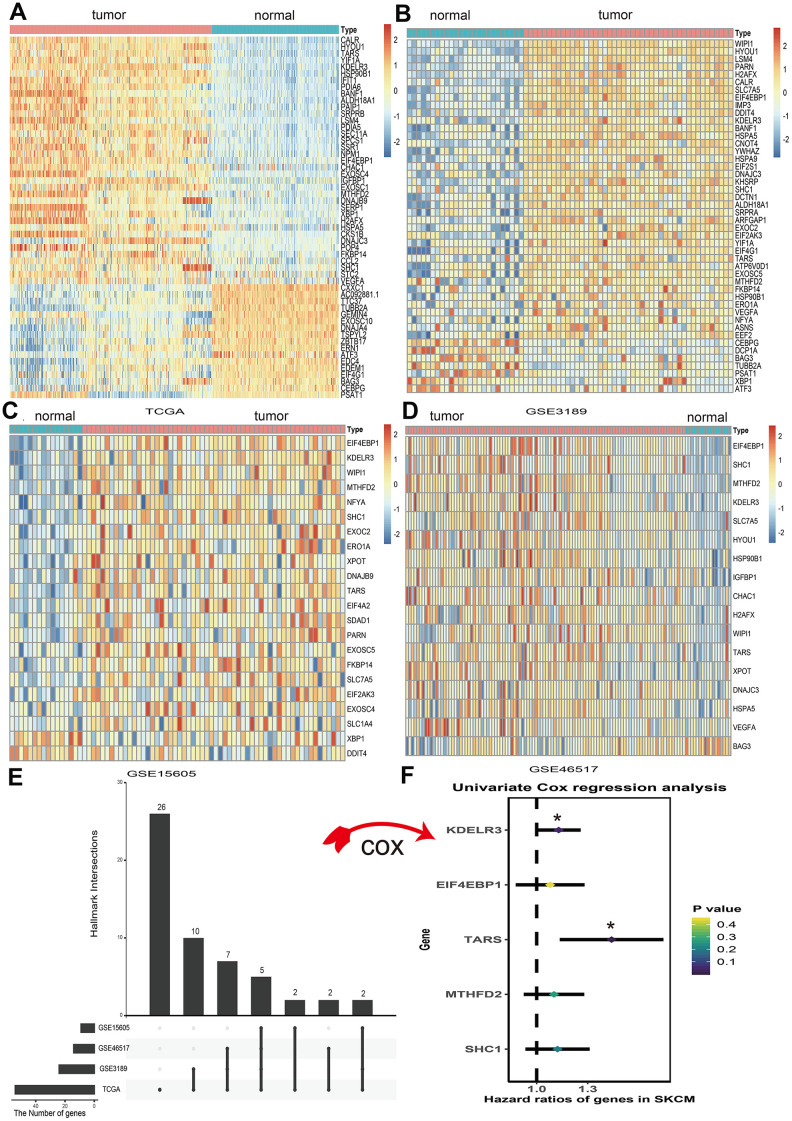
**Differential expression of unfolded protein response related genes (UPRRGs) in cutaneous melanoma tissue samples.** (**A**) Heatmap of the differentially expressed UPRRGs in TCGA dataset. (**B**) Heatmap of the differentially expressed UPRRGs in GSE3189 dataset. (**C**) Heatmap of the differentially expressed UPRRGs in GSE15605 dataset. (**D**) Heatmap of the differentially expressed UPRRGs in GSE46516 dataset. (**E**) Upset plot of differentially expressed UPRRGs in multiple datasets. The dark bar on the left of drawing represents the amount of each dataset. The dark dots in the matrix at right of drawing represent the intersections of differentially expressed UPRRGs. (**F**) Forest plots of 5 differentially expressed UPRRGs, among which only TARS and KEDLR3 significantly correlated with OS (Overall survival) in TCGA dataset. *p<0.05; **p<0.01.

**Figure 3:**
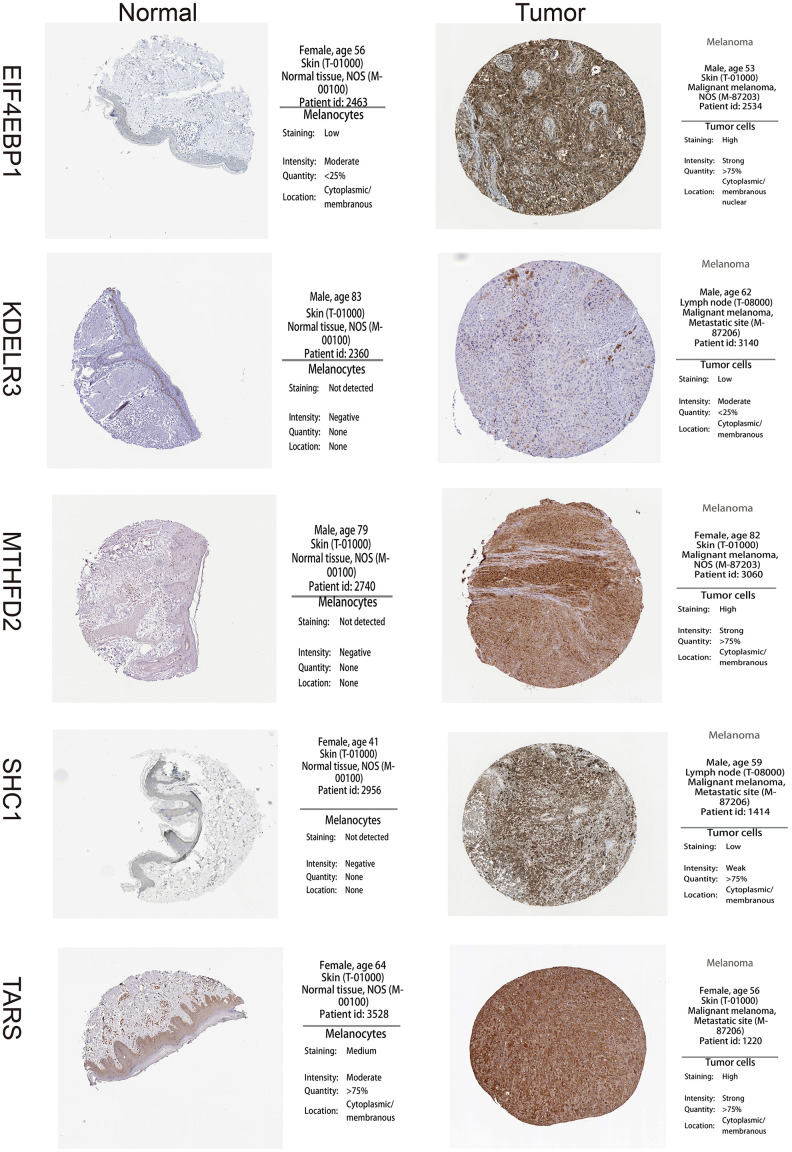
**High expression of 5 unfolded protein response related genes (UPRRGs) by immunohistochemistry in The Human Protein Atlas website.**

A total of 113 UPRRGs was obtained from the molecular signature database. Based on the differential analysis standard, 54 differentially expressed UPRRGs were selected in TCGA dataset in which 37 genes were up-regulated and 17 genes were down-regulated (Figure 2A). 46 differentially expressed UPRRGs contained 39 up-regulated and 7 down-regulated genes were distinguished in GSE3189 (Figure 2B). 22 differentially expressed UPRRGs were found in GSE15605, which including 22 up-regulated genes and 2 down-regulated genes (Figure 2C). In addition, 16 significantly up-regulated genes and 2 significantly down-regulated genes were identified in GSE46517 (Figure 2D). The overlap of differentially expressed UPRRGs among the four datasets were shown in Figure 2E. Finally, 5 differentially expressed UPRRGs were figure out for subsequent research. These genes contained KDELR3, EIF4EBP1, TARS, MTHFD2, SHC1 which all highly expressed in melanoma compared to normal skin in The Human Protein Atlas (Figure 3).

### Identification and validation of prognostic UPRRGs features

**Figure 4:**
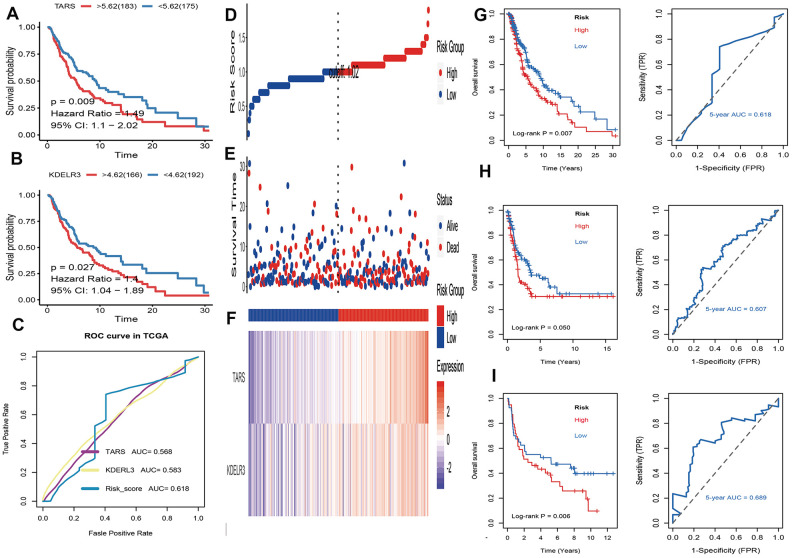
**Identification and validation of prognostic UPRRGs features for survival prediction.** (**A**) Kaplan–Meier analysis between patients in the high expression level of TARS and those in the low expression level group. (**B**) Kaplan–Meier analysis between patients in the high expression level of KEDLR3 and those in the low expression level group. (**C**) The receiver operating characteristic (ROC) curves of TARS, KEDLR3 and risk score indicators. (**D**) The distribution of risk score. the risk scores are arranged in ascending order from left to right. (**E**) Overall survival (OS) time and life status. (**F**) The prognostic UPRRGs features expression patterns for melanoma patients in TCGA dataset. (**G**) Kaplan–Meier analysis of UPRRGs features and 5 years of the receiver operating characteristic (ROC) curve in TCGA dataset. (**H**) Kaplan–Meier analysis of UPRRGs features and 5 years of the receiver operating characteristic (ROC) curve in GSE65904 dataset. (**I**) Kaplan–Meier analysis of UPRRGs features and 5 years of the receiver operating characteristic (ROC) curve in GSE54467 dataset.

Firstly, univariate cox regression analysis was applied to assess relationships between 5 differentially expressed UPRRGs and OS in TCGA dataset. The results of univariate regression for 5 differentially expressed UPRRGs were listed Table 1. Based on the selection criteria, 2 survival-related UPRRGs were seeded out (Figure 2F). Kaplan-Meier plots of 2 survival-related UPRRGs manifested that high expression of TARS (Figure 4A) and KDELR3 (Figure 4B) was associated poor survival in melanoma. Then, we used multivariate cox regression analysis to calculate coefficients for each gene and construct the risk score system. Compared the area under the curve (AUC) of TARS and KDELR3, the ROC curve for risk score was superior to KDELR3 or TARS alone (Figure 4C). The distributions of the risk scores, OS, vital status, and expression levels of corresponding UPRRGs in TCGA dataset were shown in Figure 4D–4F. Next, by applying this risk model, a risk score for each sample in TCGA dataset will be generated. Then, melanoma samples were classified into a high-risk group (n = 179) and a low-risk group (n = 179) by applying the median cut-off value of the risk scores. Kaplan-Meier curves showed that patients in high-risk group have a shorter survival time than low-risk with a log-rank test of p=0.007. To estimating the prediction power of 2 UPRRGs features, the ROC curve was drawn and 5 years of AUC was 0.618 (Figure 4G). Besides, in order to confirm the robustness of the result, verification test was conducted in GSE65904 and GSE54467 datasets. The GSE65904 and GSE54467 datasets were divided into high-risk and low-risk groups based on TCGA dataset. Kaplan-Meier curves showed that there is a significant difference between high-risk and low-risk group both in GSE65904 dataset (log-rank p=0.05) and GSE54467 dataset (log-rank p=0.006) (Figure 4H, 4I). The 5 years of AUC were 0.607 and 0.689 respectively.

**Table 1:** Univariate regression analysis for 5 differentially expressed unfolded protein response related genes.

**Gene ID**	**Gene symbol**	**HR**	**z-score**	**pvalue**
ENSG00000113407.13	TARS	1.470149	2.83026	0.004651
ENSG00000100196.10	KDELR3	1.12004	1.963382	0.049602
ENSG00000160691.18	SHC1	1.113257	1.272118	0.203331
ENSG00000065911.11	MTHFD2	1.093291	1.121635	0.262018
ENSG00000187840.4	EIF4EBP1	1.071715	0.765829	0.443778

### Correlation between UPRRGs features and clinical variables

**Figure 5:**
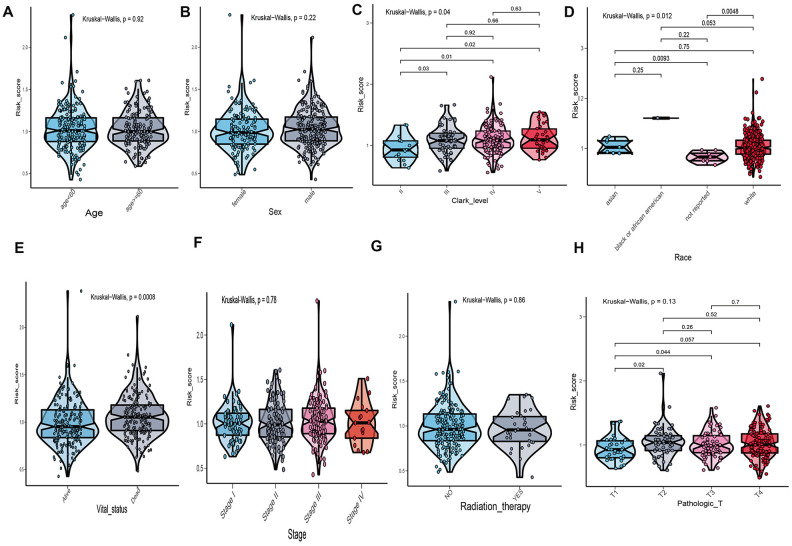
****The relationship between risk score distribution and clinical variables which include age (**A**), sex (**B**), Clark level (**C**), race (**D**), vital statu s(**E**), stage (**F**), radiation therapy (**G**) and tumor size (**H**).

The correlation between risk score of UPRRGs features and clinical variables was explored and the results of boxplot indicated that only Clark level, race and vital status were correlated with risk score (Figure 5C–5E). Other clinical variables, such as age, sex, stage and radiation therapy had no relationships with risk score (Figure 5A, 5B, 5F, 5G). As for tumor size, T2-4 had a higher risk score than T1 (Figure 5H). Hence, the UPRRGs features were associated with three clinical variables and had on effect on other clinical characteristics.

**Figure 6:**
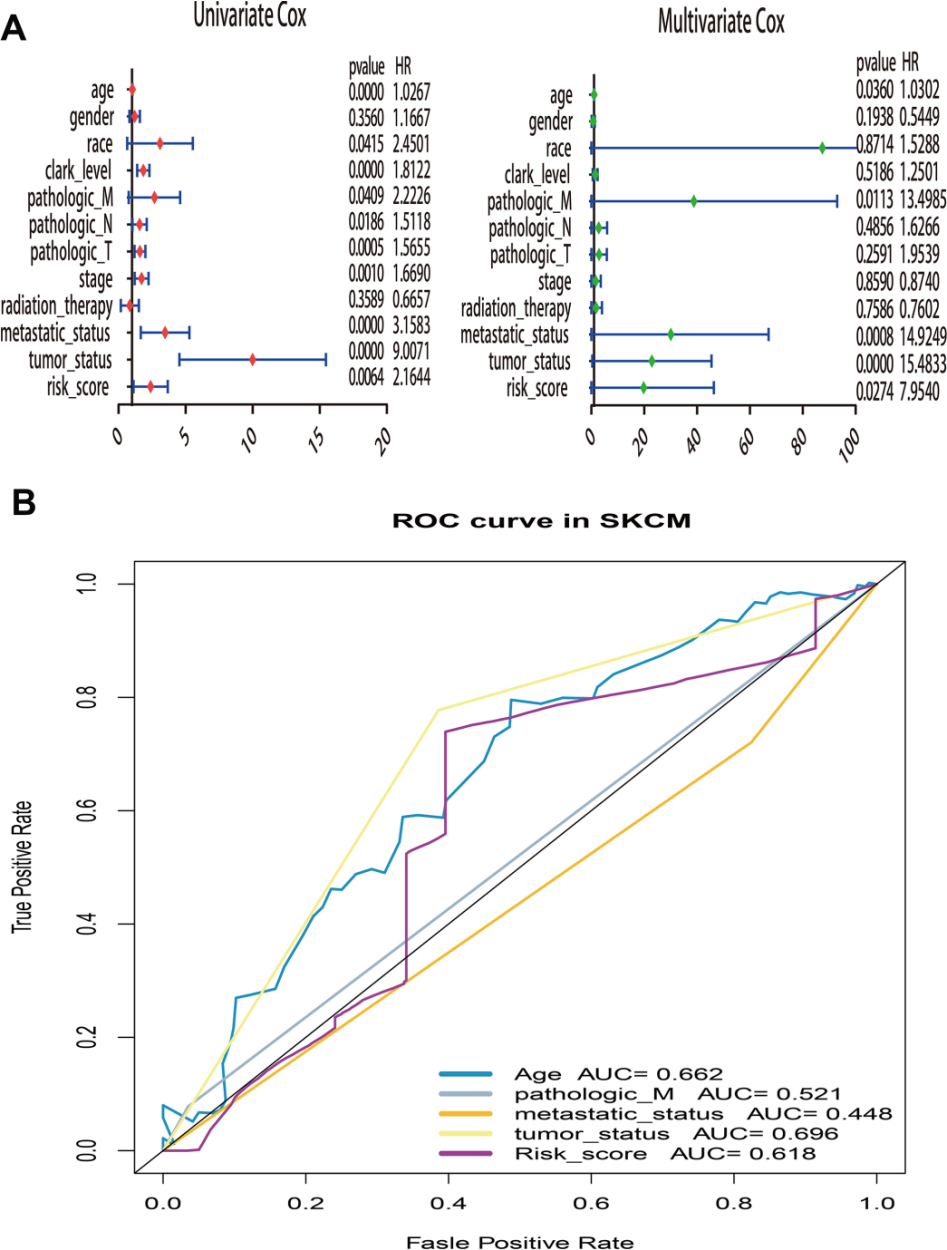
**Univariate and multivariate Cox regression analysis of risk score and clinical variables by using overall survival (OS) time in TCGA.** (**A**) Forest plots of risk score and clinical variables. (**B**) The 5 years area under the curve (AUC) of risk score and clinical variables associated with OS.

**Figure 7:**
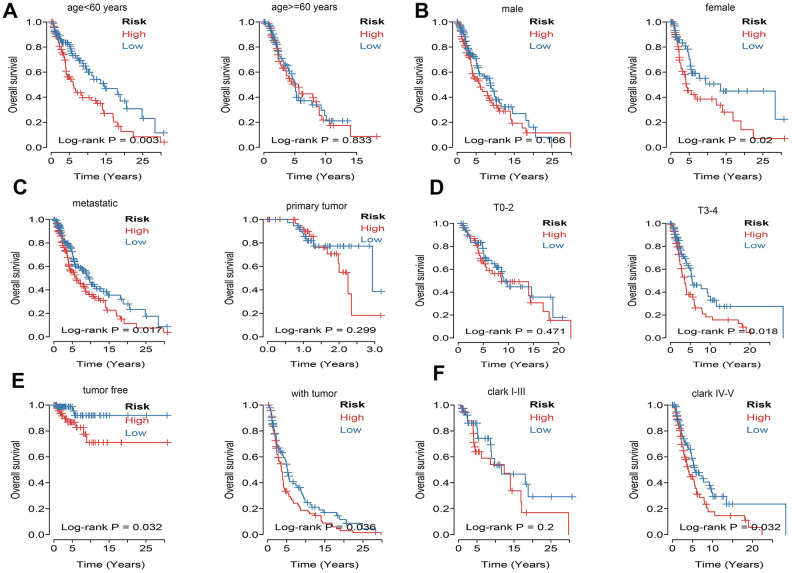
**Kaplan–Meier curve illustrates the prognostic value of risk score signature based on subgroup of different clinical variables.** (**A**) Tthe subgroup age. (**B**) The subgroup sex. (**C**) The subgroup of metastasis. (**D**) The subgroup tumor size. (**E**) The subgroup of tumor status. (**F**) The subgroup Clark level.

What’s more, to compare the prognostic value of risk score with clinical variables, univariate and multivariate logistic regression were applied. The results revealed that age, race, Clark level, TNM, stage, metastatic status, tumor status and risk sore were significantly associated with OS in univariate analysis, but only age, pathologic M, metastatic status, tumor status and risk score were significantly correlated with OS in multivariate analysis (Figure 6A). Moreover, to explore whether the risk score of UPRRGs is an independent prognostic factor, similar analyses were applied in GSE65904 and GSE54467 datasets, the results suggested that the risk score maintained significant associations with prognosis no matter in univariate multivariate regression (Table 2). The 5 years AUC of age, pathologic M, metastatic status, tumor status and risk score in TCGA were 0.662, 0.521, 0.448, 0.696 and 0.618 respectively (Figure 6B). Furthermore, in order to clarify the prognostic value of UPR features for different clinical subgroups including age, sex, metastatic status, tumor size, tumor status and Clark level were investigated. Kaplan-Meier curves showed that high-risk group in clinical subgroups such as age<60 (p=0.003), female (p=0.02), metastatic tumor (p=0.017), T3-4 (p=0.018), tumor free (p=0.032), with tumor (p=0.036) and Clark IV-V (p=0.032) had significantly shorter OS than low-risk group. However, there is no significant difference between high- and low-risk group in subgroups like age>=60 (p=0.833), male (p=0.166), primary tumor (p=0.299), T0-2 (p=0.471), Clark I-III (p=0.200) (Figure 7).

**Table 2:** Univariate and multivariate Cox regression analyses of clinicopathologic characteristics associated with survival in TCGA, GSE54467 and GSE65904 datasets.

**TCGA dataset (n=374)**	**Univariate analysis**							
	**unicox_p**	**HR**	**lower .95**	**upper .95**	**mutlicox_p**	**HR**	**lower .95**	**upper .95**
Age	0.000	1.027	1.016	1.038	0.036	1.030	1.002	1.059
Gender	0.356	1.167	0.841	1.619	0.194	0.545	0.218	1.362
Race	0.042	2.450	1.035	5.800	0.871	1.529	0.009	260.783
Clark_level	0.000	1.812	1.415	2.321	0.519	1.250	0.635	2.462
Pathologic_M	0.041	2.223	1.034	4.779	0.011	13.499	1.803	101.036
Pathologic_N	0.019	1.512	1.071	2.133	0.486	1.627	0.414	6.384
Pathologic_T	0.001	1.566	1.216	2.016	0.259	1.954	0.610	6.254
Stage	0.001	1.669	1.229	2.267	0.859	0.874	0.198	3.864
Radiation_therapy	0.359	0.666	0.279	1.588	0.759	0.760	0.132	4.369
Metastatic_status	0.000	3.158	1.842	5.415	0.001	14.925	3.083	72.242
Tumor_status	0.000	9.007	5.107	15.884	0.000	15.483	4.972	48.217
Risk score	0.006	2.164	1.242	3.772	0.027	7.954	1.260	50.213
GSE65904 (n=214)								
Age	0.797	0.998	0.985	1.012	0.778	0.998	0.984	1.012
Gender	0.169	1.335	0.885	2.016	0.296	1.248	0.824	1.891
Stage	0.348	1.140	0.867	1.500	0.558	1.087	0.822	1.439
Risk score	0.044	2.589	1.027	6.530	0.051	2.296	1.090	5.838
GSE54467 (n=79)								
Age	0.025	1.021	1.003	1.039	0.009	1.024	1.006	1.043
Gender	0.998	1.001	0.568	1.764	0.933	0.976	0.550	1.730
Stage	0.168	1.257	0.908	1.741	0.079	1.357	0.965	1.908
Risk score	0.005	2.793	1.077	4.342	0.009	2.922	1.187	5.335

### Immune microenvironment and m6A regulation between the high- and low-risk phenotype

**Figure 8:**
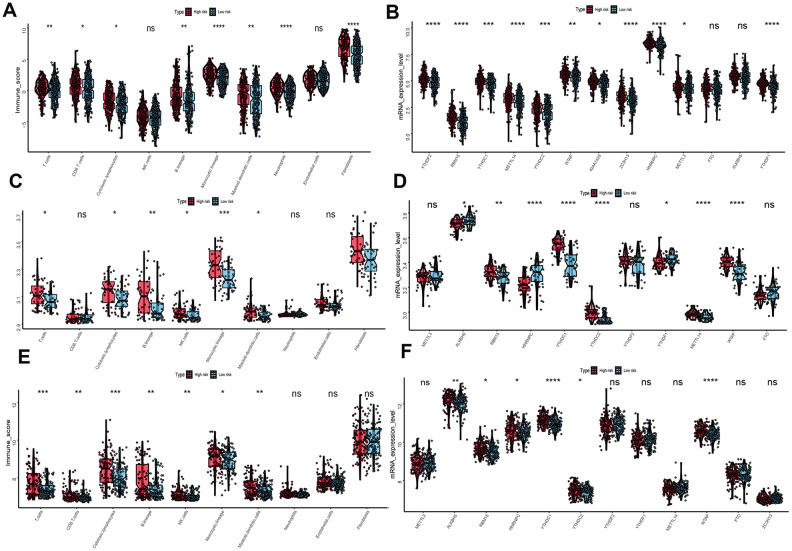
**Immune microenvironment and m6A regulation between the high- and low-risk phenotype.** (**A**) Difference immune score of 10 immune cells between the high- and low-risk melanoma patients in TCGA dataset. (**B**) Expression of N6-methyladenosine (m6A) RNA methylation regulators between the high- and low-risk melanoma patients in TCGA dataset. (**C**) Immune score distribution of 10 immune cells between the high- and low-risk group in GSE54467 dataset. (**D**) Different expression level of m6A regulators between the high- and low-risk group in GSE54467 dataset. (**E**) Immune score distribution of 10 immune cells between the high- and low-risk group in GSE65904 dataset. (**F**) Different expression level of m6A regulators between the high- and low-risk group in GSE65904 dataset. *p<0.05; **p<0.01;***p<0.001;****p<0.00001.

To evaluate the associations between UPRRGs features and immune microenvironment, subgroup analysis of immune cell individuals was performed. The boxplot showed that high-risk group of T cells, CD8 T cells, B cells, cytotoxic lymphocytes, monocytic lineage, myeloid dendritic cells, neutrophils and fibroblasts in TCGA dataset had a higher immune score than those in low risk group (Figure 8A). Similar results also found in GSE54467 (Figure 8C) and GSE65904 (Figure 8E) datasets. Besides, boxplot analysis of m6A RNA methylation regulators in TCGA showed that most of m6A regulators were differentially expressed between high- and low-risk group. The high-risk group had significantly higher expression levels of METTL14, METTL3, WTAP, KIAA1429, ZC3H13, RBM15, YTHDF2, YTHDC1, YTHDC2 and YTHDF1 (Figure 8B). The expression of METTL14, WTAP, KIAA1429, ZC3H13, RBM15, YTHDC1, YTHDC2 and YTHDF1 in GSE54467 was up-regulated in high-risk group compared to low-risk group (Figure 8D). Interestingly, Similar outcomes were found in GSE65904 dataset too (Figure 8F).

**Figure 9:**
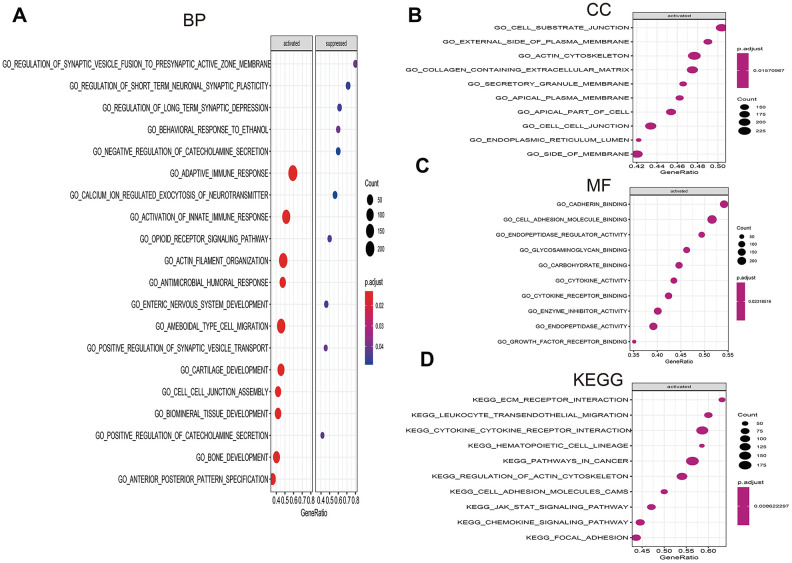
**Gene set enrichment analysis (GSEA) of high- vs. low-risk scores groups in TCGA.** (**A**) The top 10 activated pathways in biology process (BP). (**B**) The top 10 activated pathways in cellular component (CC). (**C**) The top 10 activated pathways in molecular function (MF). (**D**) The top 10 activated pathways in Kyoto Encyclopedia of Genes and Genomes (KEGG).

### Gene set enrichment analysis

To investigate the significant pathways shared by different high- and low-risk group, we performed GO and KEGG functional pathway enrichment by GSEA analysis. Based on selection standard and ordered pathways by q values. The top ten positive pathways were screen out. The biology process (BP) including adaptive immune response, calcium ion regulated exocytosis of neurotransmitter, activation of innate immune response, opioid receptor signaling pathway and so on (Figure 9A). The cellular component (CC) contained cell substrate junction, external side of plasma membrane, actin cytoskeleton, containing extracellular matrix and so forth (Figure 9B). The molecular function (MF) including cadherin binding, cell adhesion molecule binding, endopeptidase regulator activity, glycosaminoglycan binding (Figure 9C). Additionally, KEGG enrichment showed that ECM receptor interaction, leukocyte transendothelial migration, cytokine-cytokine receptor interaction, hematopoietic cell lineage and so on were positively enriched in high-risk group (Figure 9D).

## DISCUSSION

Melanoma is a most aggressive skin cancer and treatment often resistant for its genetic heterogeneity [23]. Recently, melanoma patients are growing younger and with highly metastasize and deadly threatening, which places a huge burden to thousands of people worldwide. Although genetic mutations like BRAFV600, NRAS and KIT are crucial in melanoma initiation, progression and metastasis, the pursuit of these targets often disappointed to some proportion of melanoma patients. Thus, additional prognostic events in melanoma are urgently required. Furthermore, comprehensive understanding of the cancer hallmark pathways and associated genes involved in melanoma prognosis is important for guiding treatment [24]. Therefore, to the best of our knowledge, this is the first study to systematically explore cancer hallmark pathways in melanoma based on large public datasets.

Firstly, we identified 7 hallmarks differentially activated in melanoma, which including UV response up, reactive oxygen species pathway, glycolysis, mTORC1 signal, E2F targets, unfolded protein response and DNA repair. It’s generally accepted that ultraviolet light (UV) considered as the most risk environment factor for initiation of melanoma [9]. High exposure of UV will upregulate the UV response pathways in cellular signal and then UV induced reactive oxygen species regarded as an important mutagen causes damage of skin cells is also well known [25]. Upon UV- induced reactive oxygen species pathway which also plays a crucial factor in apoptosis. To deal with protein and DNA damages caused by UV, cell will restart repair pathways, particularly glycolysis and DNA repair processes [26, 27]. In addition, previous studies demonstrated that E2F targets also conduct important functions in UV response, and associated with various biologic processes, such as DNA synthesis and replication, DNA damage and repair, cell cycle, apoptosis, self-renewal, development and differentiation, and so on [28]. Afterwards, combined with clinical survival information, we found unfolded protein response was the most significant pathway correlated with OS of melanoma patients. Obviously, the unfolded protein response has been recognized as a crucial role in tumor progression and metastasis [29, 30]. Numerous researches showed that unfolded protein response related genes (UPRRGs) are highly expressed in many cancers including colorectal, prostate, lung cancers, ovarian and breast [31–35]. Due to the vascularization and rapid proliferation of cancer cells, cancer is suffered many kinds of burden like endoplasmic reticulum stress. Meanwhile, unfolded protein response will be highly activated for rescuing the cell by removing unfolded proteins [36, 37]. In melanoma patients, same results also found that unfolded protein response is positively associated with tumor progression, size and poor prognosis of patients [38, 39]. Therefore, it is undoubted that the unfolded protein response executes significant functions in melanoma.

In this research, we further distinguished 5 differentially expressed UPRRGs (KDELR3, EIF4EBP1, TARS, MTHFD2 and SHC1), which also highly expressed in melanoma at the protein level. Among the 5 UPRRGs, TARS and KDELR3 are correlated with OS and used to developed a robust UPRRGs feature which also was validated in another two independent datasets. Kaplan-Meier plots showed that UPRRGs feature revealed a good survival prediction of melanomas. Next, the Kaplan–Meier plots in clinical subgroups manifested that, especially in subgroups like age<60, female, metastatic, T3-4, and Clark IV-V, there were significant differences between prognosis in the low- and high-risk group. These results showed that the patients in the high-risk groups always survive shorter than those in low-risk groups, which indicated that the identified UPRRGs feature is more suitable to predict melanoma patients with younger, metastatic and high Clark level. In addition, the risk score of UPRRGs feature was only related to Clark level, race and vital status and had on effect on other clinical variables. Besides, the univariate and multivariate regression analysis indicated that the risk score of UPRRGs feature could be regard as an independent prognostic model in melanoma. Notably, compared with the traditional clinical characteristics, our UPRRGs feature can achieved similar accuracy of other clinical indicators (eg. age, tumor status, metastatic status and pathologic M).

As we known, m6A modification takes a crucial role in tumor initiation and cancer progression and recurrence [40]. RNA methyltransferases (such as METTL14, METTL3 and TAP), the demethylases(such as ALKBH5 and FTO), and the binding proteins (such as YTHDF2 and YTHDF1) are often upregulated in a variety of human cancer types to increase the expression of oncogenes and oncoproteins [41]. In our research, the most expression of m6A regulators including METTL14, METTL3, WTAP, KIAA1429, ZC3H13, RBM15, YTHDF2, YTHDC1, YTHDC2 and YTHDF1 were highly expressed in high-risk of melanoma patients, and hence we have enough reasons to believe that our UPRRGs feature closely correlated with the prognosis of melanoma. What’s more, melanoma patients in high-risk group had higher immune infiltration than low-risk group. Recently, many researches had been proved that the immune environment intimately correlated with melanoma initiation and development [42, 43]. The uneven distribution of immune cells was also positively associated with prognosis of cancer patients.

To better understanding the underlying biological mechanism in high-risk group, we also applied GSEA method to analyze the potential signaling pathways enriched in high-risk group. The results showed adaptive and innate immune response as major biology process activated in high-risk group, which was consistent with our previous findings. Cellular component mainly enriched in extracellular matrix such as cell substrate junction, external side of plasma membrane, actin cytoskeleton and cell to cell junction, which are pivotal in cancer cell invasion [44]. Molecular function primarily enriched in cell adhesion molecule including cadherin binding, cell adhesion molecule binding, endopeptidase regulator activity and glycosaminoglycan binding, which closely associated with the regulation of tumor progression [45–47]. Moreover, KEGG pathway analysis showed high-risk group mainly related to ECM-receptor interaction and leukocyte transendothelial migration. ECM-receptor interaction pathway is important in metastasis [48]. The significance of the ECM-receptor interaction pathway implied the interaction between tumor cell and environment are very dynamic [49].

Although we identify some significant cancer hallmarks and unfolded protein response related genes for prognostic of Melanoma. However, our research still has some limitations. Firstly, our analysis was implemented based on existing data using bioinformatics method. Our findings have not been proved by experiments or patient tissue. This was the main weak point of this study. Besides, the sample size of our study is limited and need further research with large sample size.

## CONCLUSIONS

In summary, our study identified several hallmark pathways and prognostic UPRRGs feature in melanoma. The biomarker and pathways supply a more simple and accurate prediction for the prognosis of melanoma in clinical application. Furthermore, investigations are needed to verify the accuracy for estimating prognoses and to test its clinical utility in patient management.

## MATERIALS AND METHODS

### Data collection and procession

The transcriptome profiles of RNA sequencing data of cutaneous melanoma as well as clinical information were obtained from the Xena Public Data Hubs (http://xena.ucsc.edu) and GEO database (https://www.ncbi.nlm.nih.gov/geo). Gene expression profiles GSE3189, GSE15605 and GSE46517 were downloaded from GEO database. The GSE3189 dataset contained 70 samples, including 7 normal skin, 18 nevi and 45 melanoma samples. GSE15605 had 16 normal skin samples and 58 melanoma samples. GSE46517 included 121 samples which consist of 9 nevus samples, 8 normal skin and 104 melanoma samples. The TCGA expression profile of cutaneous melanoma were downloaded from the Xena Public Data Hubs, which contained 372 melanoma samples and 233 healthy skin tissue samples. The characteristics of datasets were summarized in Table 3. The raw data were processed by applying R software. Firstly, the probe IDs were annotated according to the annotation information of platform. For the same gene corresponding to multiple IDs, the max expression value will figure out to represent the gene expression level. Next, genes with a variance of 0 will be excluded for its tiny expression level. Finally, the raw matrix data were normalized by log2(x+1) conversion.

**Table 3:** Summary of datasets used in this research. NA means not available.

**Data set**	**Platform**	**Sample size (tumor/normal)**	**Median age (year)**	**Sex (male%)**	**Metastasis(%)**
TCGA-SKCM	Illumina HiSeqV2	605 (372/233)	58.15	62.05	77.78
GSE3189	Affymetrix Human Genome U133A Array	70 (45/25)	65.51	51.1	NA
GSE46517	Affymetrix Human Genome U133A Array	121 (104 /17)	58.19	72.54	70.19
GSE15605	Affymetrix Human Genome U133 Plus 2.0 Array	74 (58/16)	59.27	65.51	20.69
GSE65904	Illumina HumanHT-12 V4.0	214 (214/0)	62.35	57.94	NA
GSE54467	Illumina HumanWG-6 v3.0 expression beadchip	79 (79/0)	56.15	63.29	12.65

### Gene set variation analysis (GSVA)

To explore the differential activities of pathways between melanoma and normal sample, A total of 50 cancer hallmark pathways were obtained from the molecular signature database (MSigDB). For the overlap of genes in each pathway will be removed to ensure very pathway gene set consist of unique genes. Afterwards, most hallmark pathways remained more than 70% of their related genes. Then gene set variation analysis was used to evaluate the common pathways shared in tumor and normal groups. The GSVA scores of each pathway for each sample were calculated by using the R package (“GSVA”) and “Limma” package was applied to explore the differential activities of pathways. The | t value of GSVA score | ≥ 1 was regarded as the cutoff criterion for differential activities of pathways [50].

### Evaluation of the prognose of cancer hallmark pathways

To evaluate the prognostic cancer hallmark pathways in melanoma, the common pathways enriched in tumor samples were identified from the four microarray datasets (GSE3189, GSE15605, GSE46517 and TCGA). Then, the cancer hallmark pathways will be regarded as continuous variables. The associations between pathways and overall survival (OS) time were assessed in TCGA dataset. Univariate Cox analyses were used to distinguish the prognostic pathways (p values <0.05). Ultimately, only two cancer hallmark pathways included DNA repair and unfolded protein response (UPR) significantly correlated with OS in melanoma. Especially, the unfolded protein response considered as the most significantly pathway was screen out and the unfolded protein response related genes (UPRRGs) were extracted from the gene set of unfolded protein response for subsequently analysis.

### Differential analysis of UPRRGs

Firstly, the gene expression levels of UPRRGs were extracted from the four datasets (GSE3189, GSE15605, GSE46517 and TCGA). These datasets were classified into tumor group and normal group. Next, the differential analysis was performed to identified differentially expressed UPRRGs by conducted “Limma” method in R software. The cutoff standard was The |log 2 FC| ≥ 0.5 and p values <0.05. Then, the Upset plot analysis was used to explore the overlap of differentially expressed UPRRGs among these datasets. In addition, these genes will further be validated in The Human Protein Atlas database.

### Identification and validation of UPR features

The association between the overlap of differentially expressed UPRRGs and OS of melanoma patients in TCGA was analyzed. Univariate cox analyses were performed to select the prognostic differentially expressed UPRRGs. Then, multivariate cox regression analysis method was applied to construct prognostic features with identified prognostic UPRRGs for the risk formula and the risk score is generated as follows: Risk score = $\sum_{i=1}^{N}(coef_{i}\times expr_{i})$, in which N means the number of feature genes, expr_i_ means the expression level of genes and coef_i_ means regression coefficient. The risk score of each sample in TCGA dataset was estimated and the patients were accordingly classified into high- and low-risk group by median cutoff. To compare the differences between high- and low-risk group, Kaplan–Meier survival curves were drawn and significance was calculated by log-rank tests. The area under the curve (AUC) of Receiver operating characteristic curves (ROC) was used to evaluate the 5-year overall survival predictive accuracy of the model. Besides, in order to test the robustness of the result, these UPR features were further verified in another two independent datasets (GSE65904 and GSE54467) which were downloaded from GEO database.

### Evaluation of relationship between UPR features and clinical variables

In order to clarify the relationship between risk score of UPR features distribution and clinicopathologic characteristics, the subgroup analysis of clinical variables included age, sex, race, stage, tumor size, vital status, Clark level and radiation therapy were performed. Moreover, in order to compare the prognostic value between the risk score and clinical variables. The univariate and multivariate cox logistic regression were carried out to define prognostic factors in multiple datasets (TCGA, GSE65904 and GSE54467). Next, these melanoma patients were stratified into subgroups based on their clinical variables, such as age (<60 or >=60), sex (male or female), metastatic status (metastatic or primary), tumor size (T0-2 or T3-4), tumor status (tumor free or with tumor) and Clark level (I-III or IV-V). Kaplan–Meier plots were used to explore the prognostic value of UPR features in different clinical subgroups.

### Evaluation of association between UPR features and immune microenvironment

To explore the relationship between UPR features and immune microenvironment in melanoma, “MCPcounter” package in R was applied to specifically discriminate 8 human immune cells and 2 stromal cells which including T cells, CD8 T cells, B cells, cytotoxic lymphocytes, natural killer (NK) cells, monocytic lineage, myeloid dendritic cells, neutrophils, endothelial cells and fibroblasts. Next, these immune and stromal cells were divided into high- and low-risk groups according the risk score of UPR features and then subgroup analysis of these cells were performed.

### Evaluation of association between UPR features and N6-methyladenosine (m6A) RNA methylation regulator

m6A RNA methylation regulators have been proven to play important regulatory roles in tumor initiation and progression, thus the difference of m6A RNA methylation regulator expression between high- and low-risk groups were also investigated. Firstly, thirteen m6A RNA methylation regulators selected from previously published articles, which included ALKBH5, FTO, METTL14, METTL3, WTAP, KIAA1429, ZC3H13, RBM15, YTHDF2, YTHDC1, YTHDC2 and YTHDF1 [51, 52]. Next, the RNA expression data of thirteen m6A regulators in three datasets (TCGA, GSE65904 and GSE54467) was extracted and divided into high- and low-risk groups. Finally, subgroup analysis of these genes was also conducted.

### Gene set enrichment analysis

In order to explore the different signaling pathways between the low- and high-risk groups, Gene Set Enrichment Analysis (GSEA) was conducted by “clusterProfiler” package in R software. Firstly, the differential analysis of all genes between low- and high-risk groups were generated and these genes were ordered by the value of log2 fold change. Then GSEA was performed to investigate the signaling pathways correlated with different subgroups of melanoma. The q value<0.05 was applied to selected the significant pathways enriched in each phenotype.

### Statistical analysis

All statistical analyses were conducted using R package (v.3.6.0) and corresponding packages.

### Data availability

Data generated and/or analyzed during the current study can be obtained from the corresponding author on reasonable request.
